# Determining the protocol requirements of in-home cat food digestibility testing

**DOI:** 10.3389/fvets.2023.1129775

**Published:** 2023-05-11

**Authors:** E. Bos, W. H. Hendriks, B. Beerda, G. Bosch

**Affiliations:** ^1^Animal Nutrition Group, Wageningen University & Research, WD Wageningen, Netherlands; ^2^Behavioral Ecology Group, Wageningen University & Research, WD Wageningen, Netherlands

**Keywords:** adaptation, fecal collection, sample size, protocol requirements, in-home test

## Abstract

In-home cat food digestibility testing has the potential to yield data that are highly representative of the pet population for which the food is intended. However, no standardized and validated in-home digestibility test protocols are currently available. Such protocols for in-home testing should address key factors that explain variation in cat food digestibility values and here we investigated the required period of adaptation, fecal collection and sample sizes. Thirty privately-owned indoor housed cats of various breeds (20♀ 10♂, 5.9 ± 3.9 yr, 4.5 ± 1.3 kg) received a relatively low and high digestible complete dry extruded food with the marker titanium (Ti) dioxide. Foods were given in a cross-over design of 2 periods of 8 consecutive days each. Owners collected feces daily for the determination of daily fecal Ti concentrations and digestibility of dry matter, crude protein, crude fat, and gross energy. Data originating from 26 cats were analyzed as mixed models and broken line regressions to investigate the required adaptation and fecal collection period. Bootstrap sampling was used to assess the impact of increasing the number of fecal collection days and sample size on the precision of the digestibility estimates. Feces were collected on 347 out of 416 study days (16 days/cat; 26 cats), implying the necessity for multiple collection days to account for cats not defecating every day. Cats showed stable fecal marker concentrations from day 2 onwards when fed the low digestible food and from 3 onwards when fed the high digestible food. Digestibility values were stable from day 1, 2 or 3 onwards, depending on the test food and nutrient. Increasing the number of fecal collection days from 1 to 6 days did not result in more precise digestibility estimates, whereas increasing the number of animals from 5 to 25 cats did. For future in-home digestibility tests of cat food, the findings support a minimum of 2 adaptation days and 3 fecal collection days. Appropriate sample sizes depend on the test food, the nutrient of interest, and the acceptable margin of error. The findings of this study support the protocol development for future in-home digestibility testing of cat foods.

## 1. Introduction

Commercial cat foods are the main source of nutrients of Western pet cats (1, 2), making the nutritional quality of such foods of paramount importance. To evaluate the quality of formulations, ingredients, and processing technologies, pet food companies routinely conduct digestibility testing especially on the final product. Digestibility testing of cat foods is almost exclusively conducted at dedicated facilities that employ a limited number of cats housed under standardized conditions. The latter differs greatly from the actual living conditions of cats in households, making the results on food digestibility being less representative for the pet cat population for which the foods are intended. Findings are expected to be more representative if these are obtained with pet cats housed at their home environment, i.e., ‘in-home’ (3, 4). In-home testing can not only provide information on nutrient digestibility in cats with different characteristics (e.g., sex, neuter status, age, breed, food history) and living conditions (e.g., housing, eating pattern, activity), but also on important parameters related to food quality as perceived by owners. Protocol requirements for in-home cat food digestibility testing, such as study duration (adaptation and fecal collection) and study population (number of cats and cat characteristics) likely differ from those commonly used (5, 6) in dedicated facilities. For example, the increased variety in test subjects and relatively uncontrolled test conditions will impact the precision of the determined digestibility values.

The current protocol requirements for experimental studies on cat food digestibility provided by AAFCO (5) and FEDIAF (6) include a minimum of six healthy, fully grown cats over 1 year of age, individually housed for 10 days with 5 days of adaptation and 5 days of fecal collection. The required length of the adaptation period to assess digestibility values may vary between nutrients and energy. Digestibility values of four cats measured over days 4–7, 8–14 and 15–21 (pooled faecal samples per cat) were similar for dry matter (DM), energy, fat and N-free extract (7). A longer adaptation period is, however, supported by differences for crude protein digestibility and wet feces output for days 8–14 compared to days 15–21 (7). The optimal period of adaptation warrants further investigation as in a recent study with dogs (*n* = 53) it was found that a 1-day adaptation period suffices to reach constant digestibility values (8). The digestive system adapts rapidly and digestive enzyme activity and microbiota composition changes within a few hours after changes in the amount and type of dietary protein, carbohydrate or lipid (9–11). Having several fecal collection days allows to smooth out day-to-day variation in fecal composition, and the pooling of fecal samples of pigs has been shown to result in more precise digestibility estimates (12). The day-to-day variation in digestibility estimates did not show in the group of pet dogs and the inclusion of multiple days did not increase the precision of digestibility estimates (8).

The use of an indigestible marker is a more practical approach than quantitative feces collection when conducting in-home digestibility studies (3). In the case an indigestible marker is used, the adaptation period is not only important to allow the animal’s digestive system to adapt to the test food (9), but also to ensure a constant marker excretion in the feces (13). The time to reach the latter is determined by the gastrointestinal transit time, which might vary between cats in-home. Even when controlling for diet, environment and genetic background, individual cats show high variability in gastrointestinal transit time (14) and vary between young (26.5 ± 5.8 h) and older cats (35.7 ± 14.1 h) (15), and between fasted (28.9 h, range 18.4–90.9 h) and fed state (46.6 h, range 15.4–109.4 h) (14). Housing conditions, meal size, and meal frequency are all likely to impact gastrointestinal transit time as these influence gastric emptying rate (16, 17). As in-home digestibility testing is conducted under less controlled conditions, the greater variability compared to testing in dedicated feline facilities would require a larger number of cats to achieve the same level of precision as obtained with six cats prescribed within the AAFCO (5) and FEDIAF (6) protocols. Factors such as age, body condition, food history, feeding level and owner (non) compliance may impact on digestibility estimates (18–27), and in a heterogenous study population of pet cats such factors raise variation in food digestibility estimates. The consequences for minimal sample sizes for in-home studies are yet to be determined.

The present in-home study assesses the degree of variation in food digestibility values in privately-owned cats across 8 days, with the aim to determine the minimal period of adaptation and fecal collection, as well as the required number of cats. The findings support protocols for in-home cat food digestibility testing.

## 2. Experimental methods

### 2.1. Study design

In-home digestibility tests were conducted with privately-owned cats in which owners fed their cat two different dry extruded foods and collected their cat’s feces on a daily basis. A cross-over design was used with two consecutive 8-day feeding periods and the start food was alternated across participants based on the order of entering the study (i.e., owner 1 Food A, owner 2 Food B, owner 3 Food A, owner 4 Food B, etc.). Participation in the study occurred from February to May 2021 and participants started the in-home digestibility study on different days during this period.

The study was approved by the Animal Welfare Body of Wageningen University (Wageningen, Netherlands) and did not qualify as an “animal experiment” according to the Dutch Experiments on Animals Act (2014). An inform consent was signed by the cat owners before the start of the study and the surveys used in this study adhered to the guidelines for privacy and data handling of Wageningen University & Research. The completion of surveys did not interfere significantly with normal daily life of the cat owners, and the questions were not psychologically burdening, thereby, exempting the surveys from approval by the ethics committee according to the guidelines of Wageningen University Medical Ethics Review Committee (Medisch Ethische Toetsingscommissie van Wageningen University, METC-WU).

### 2.2. Participants

Dutch cat owners were recruited through online advertisements. Candidate participants completed an online questionnaire (Microsoft Forms) on predominantly demographics and cat characteristics. Cat owners were eligible when they were willing to provide the test food as the sole source of nutrition to their cat, collect the cat’s feces and control both its individual food intake and place of defecation through the use of a dedicated litterbox. Mainly owners of single- and indoor-housed cats met these criteria. Cats were eligible when older than 1 year, not pregnant or lactating and healthy (no medication, no intestinal upsets over the past 3 months, free of previously diagnosed chronic diseases, no food allergies or intolerances). Cats described by the owner as a “difficult eater” received a sample of the test food (1 daily portion) prior to the study and entered the study only when readily accepting the test food. Owners rated their cat’s pickiness in the online questionnaire on a 5-point scale from a very difficult eater (score 1) to and an easy eater (score 5) with scores <3 being identified a difficult eater. The cat owners that satisfied the inclusion criteria entered the study and the number of cats entering the study was determined by the available resources needed to run the study (e.g., time, funding, logistics, sample processing, chemical analyzes).

The participants received a brochure containing information about the study and received further explanation in person about how to perform the tasks during a visit when materials were delivered. The latter included daily food portions, lime and quartz sand cat litter (Catsan Hygiëne Plus, Catsan™), feces collection bags, feces information bags, a freezer container, a mini freezer (Primo DV2-WS, Primo Elektro, Herentals, Belgium) if requested, and a diary. The diary included the Waltham Feces Scoring Chart (28) and owners were instructed on how to score feces for consistency. During the study, owners were contacted by the researcher at least once by email and could contact the researcher by email and/or phone any time.

### 2.3. Foods and feeding

The two test foods were commercial dry extruded cat foods (Jonker Petfood BV, Waalwijk, Netherlands) that differed in ingredient quality and inclusion and in nutrient composition such that it established a contrast in nutrient digestibility. Food formulations (Table 1) met the nutritional guidelines of FEDIAF for adult cats (6) and included TiO_2_ (Hombitan FG, Venator Germany GmbH, Duisburg, Germany) as an indigestible marker. The particle size distribution of TiO_2_ was determined by the Mastersizer 3,000 (Malvern Panalytical BV, Almelo, Netherlands) and was devoid of nanoparticles [<100 nm (29)]. Dry ingredients and the marker were mixed for 60 s in a paddle shift mixer (Forberg F60, Forberg International AS, Oslo, Norway), followed by extrusion using a co-rotating double screw extruder (Baker Perkins MF50, Baker Perkins, Manor Drive, United Kingdom), oven-drying at 45°C overnight, vacuum coating with pre-heated (60°C) poultry fat and liquid digest, and mixing (Dinnissen 305, Dinnissen BV, Sevenum, Netherlands) with powder digest at the research facilities of Wageningen University & Research (Wageningen, Netherlands).

**Table 1:** Ingredient composition and analyzed chemical composition and energy contents of the relatively high (Food A) and low (Food B) digestible dry extruded cat foods.

Component	Food A	Food B
Ingredient composition (g/kg)		
Wheat	–	350
Wheat-semolina	–	150
Rice	260	–
Linseed oil	5.00	–
Beet pulp	30.0	–
Poultry fat	109	57.9
Salmon oil	10.0	–
Digest	30.0	7.49
Fish meal	310	–
Greaves	–	140
Meat bone meal	–	120
Premix	14.0	12.0
Barley	–	100
Keratin protein	–	50.0
Potato	184	–
Choline chloride	1.00	2.50
Fibers	5.00	10.0
Rice protein	41.5	–
Titanium dioxide	1.00	1.00
Chemical constituents (g/kg as-is)		
Dry matter	931	937
Crude ash	65.4	63.1
Organic matter	935	937
Nitrogen	49.3	47.4
Crude protein	308	297
Crude fat	109	149
Starch	348	263
Total dietary fiber	103	191
Titanium	0.55	0.53
Energy (MJ/kg as-is)		
Gross energy	19.5	20.5
Metabolizable energy*	14.3	13.4

* Calculated using NRC, 2006 (66).

The foods were formulated to contain either 16.0 MJ/kg ME (food A) or 14.2 MJ/kg ME (food B) and were fed at maintenance energy requirements (314 kJ × kg BW^0.67^; FEDIAF, 2021). Feeding levels were discussed with the owner prior, or when requested, during the study and adjusted where appropriate. The cats were fed following the feeding schedule they were used to (i.e., meals or *ad libitum*), but were all instructed to start the study in the morning on the first day. Cat owners were provided with daily food portions, instructed to only provide the test food to their cat, and to carefully collect and store leftovers each day. Water was instructed to be provided *ad libitum*.

### 2.4. Feces and data collection

The cat owners were asked to daily collect their cat’s feces from the litter box with as little contamination as possible. Due to feasibility reasons, owners collected feces mostly once a day. The collection might not have been directly after their cat’s defecation, resulting in fresh and non-fresh feces samples being collected within a 24 h timeframe each day. Single feces samples were collected with the collection bag and placed in an information bag with a label containing the cat’s name and the collection date and time. Storage occurred at −18°C in a freezer of the owner or one that was temporarily provided. Feces were translocated to Wageningen University & Research within 2 weeks after an owner had completed the study and stored at −20°C pending further processing and chemical analyzes.

Cat owners were requested to fill in a daily diary providing information on consumption of the test foods and (accidental) other items, as well as feces characteristics (number of defecations; feces consistency score according to the Waltham Feces Scoring Chart (28), with score 1 indicating hard and dry feces and score 5 indicating watery diarrhea; additional particularities).

### 2.5. Chemical analyzes and calculations

Feces were pooled per day (including 24 h post first food consumption), resulting in maximum eight samples per cat per period/food. Feces were oven-dried at 60°C to reach a constant weight. Water loss after oven-drying was not used to calculate dry matter of the fresh feces as cat litter was still included, which renders such data inaccurate. After drying, all visible cat litter granules and cat hairs were manually removed and the cleaned feces were ground to pass a 1-mm sieve in an ultra-centrifugal mill (ZM100, Retsch B.V., Ochten, Netherlands). Fecal samples were analyzed by near-infrared reflectance spectroscopy (NIRS; Anadis Instruments Benelux BV & Nirvention BV, Almere, Netherlands), with the NIRS being calibrated by chemical analyzes of a subset of 50 feces samples obtained in this study. Samples of feces and foods were analyzed in duplicate for dry matter (30) (DM), nitrogen (31) (N), crude fat (32) (Cfat), and gross energy (33) (GE). Crude protein (CP) has been calculated as N × 6.25. Food samples were also analyzed for total dietary fiber (34). Ti concentrations in foods and all fecal samples were determined using inductively coupled plasma-optical emission spectrometry (ICP-OES, Iris intrepid II XSP, Thermo Fisher Scientific, Inc.) after destruction with H_2_SO_4_ using a microwave digestion system (MARS 6, CEM Corporation, Matthews NC, Unites States).

Apparent fecal nutrient digestibility was calculated as described elsewhere (5, 6):


$$Nutrientdigestibility\;\left(\%\right)=100-\frac{\left[Nut_{feces}\right]\times \left[Ti_{food}\right]}{\left[Nut_{food}\right]\times \left[Ti_{feces}\right]}\times 100\%$$

where Nut_feces_, Nut_food,_ Ti_feces_ and Ti_food_ are the nutrient content (% DM) and Ti content (% DM) of feces and food, respectively.

### 2.6. Data processing and statistical analyzes

Negative digestibility values, which were predominantly observed during day 1, were omitted from the dataset (n = 49 out of 1,648 records). To model how nutrient digestibility values varied with the inclusion of a different number of fecal collection days, the digestibility values across subsequent fecal collection days were averaged. A new dataset was created including calculated pooled digestibility values per cat and food across 2 to 6 collection days.

All data were statistically analyzed using SAS (v. 9.4; SAS Institute, Cary, NC). The plateau (day) of time functions of fecal Ti concentrations as well as digestibility values were estimated with breakpoints in linear broken-line regressions (35) using the NLIN procedure. The broken-line function was as follows:

$Y=a+b\times x-b\times 0.01\times \log\;\left(1+e^{\left(c-x\right)/0.01}\right)$

where Y is the dependent variable, a is the starting value when x= 0, x is the day, b is the slope when x<c, and c is the breakpoint. Parameters of the broken-line function were estimated for the study cat population as well as per individual cat, and separately for each feeding period (1, 2) and for each food (A, B). These separate analyzes were considered more accurate and informative as feeding periods 1 and 2 differed in the food that cats received before the period started, with the owner’s choice of food in period 1 (no Ti) vs. the experimental food in period 2, and this was expected to show in the starting values of fecal Ti concentrations and digestibility values. Due to missing values (e.g., because a cat did not defecate every day), the estimation of individual breakpoints in broken-line functions was not possible for every cat during each period and/or food and these cats were omitted from the dataset.

The required lengths of the adaptation and fecal collection periods were also determined from time-dependent variation in fecal Ti concentrations and digestibility values as assessed by a repeated measures ANOVA using the Proc MIXED procedure. Again, time effects were analyzed separately for each feeding period (1, 2) and for each food (A, B). Day was used as a REPEATED model statement (36) using a first-order autoregressive covariance structure [AR(1)] (37) and the model:


$$Y=\mu +D_{i}+\varepsilon_{i}$$

where *Y* is the dependent variable, *μ* is the average intercept, *D_i_* is day *i*, and *ε_i_* is the error term. Differences were considered significant at a probability <0.05, with posthoc pairwise comparisons were analyzed using the Tukey test. The consequences for the precision of digestibility estimates of the number of fecal collection days in combination with sample sizes were assessed using bootstrap sampling, including 10,000 replicates.

The effects of experimental factors (food and period) and cat characteristics on digestibility values were analyzed by repeated measures ANOVA in Proc MIXED, using an entire dataset across feeding periods and foods, but including only the study days after a constant marker excretion in the feces was reached (on cat population level). The statistical model was optimized for explaining variance in a dependent variable (DM, CP, Cfat, and GE digestibility) by using stepwise regression with the GLMSELECT procedure and the Schwarz Bayesian information criteria. The independent variables were the test food (A, B), period (1, 2), sex (female, male), neuter status (intact, neutered), age, weight and the two-way interactions between any two variables.

## 3. Results

### 3.1. Participants and owner compliance

This in-home digestibility study was started with 30 cats from 29 owners (20♀ 10♂, 5.9 ± 3.9 yr, 4.5 ± 1.3 kg; Supplementary Figure S1), of which 27 cats from 26 owners completed the study. Dropouts included cats that did not eat both foods (*n* = 2) and one owner who did not comply to the study protocol (*n* = 1). Four owners indicated that their cat had to get used to the provided cat litter and two of those switched back to their own cat litter during the study.

The owners reported that other items than the test food were consumed by 9 cats on 17 study days out of the total 432 records from 27 cats, including human food (*n* = 4 cats, on 5 days), cat food (*n* = 5 cats, on 6 days), cat treats (*n* = 2 cats, on 4 days) and dog food (*n* = 1 cat, on 2 days). Fecal samples were not available for 76 days, mainly because of no defecation (*n* = 72) and due to non-compliance (*n* = 4 days, by two owners).

Two fecal samples of one cat were too contaminated with cat litter and were excluded from chemical analyzes. Data for one cat were rejected for reasons of deviating fecal Ti concentrations (defined as >2× std. dev from mean) and suspected non-compliance of the cat owner, rendering data on 26 cats for statistical analyzes (16♀ 10♂, 5.5 ± 4.0 yr, 4.4 ± 1.2 kg; Supplementary Table S1).

### 3.2. Food transitions

The transitions between foods, from a cat’s usual food to one of the two experimental foods and between experimental foods, caused minimal digestive discomfort in the cats. Vomiting was reported by owners on day 1 (*n* = 1; Food B), 5 (*n* = 1; Food B), 7 (*n* = 1; Food B), and 8 (*n* = 1; Food A). Extreme fecal consistency scores were rare (scores 1 or 5, *n* = 6), scores of 1.5 were recorded 109 out of 453 times, and scores 4.5 were not reported.

### 3.3. Variation in fecal Ti concentrations across days

**Figure 1:**
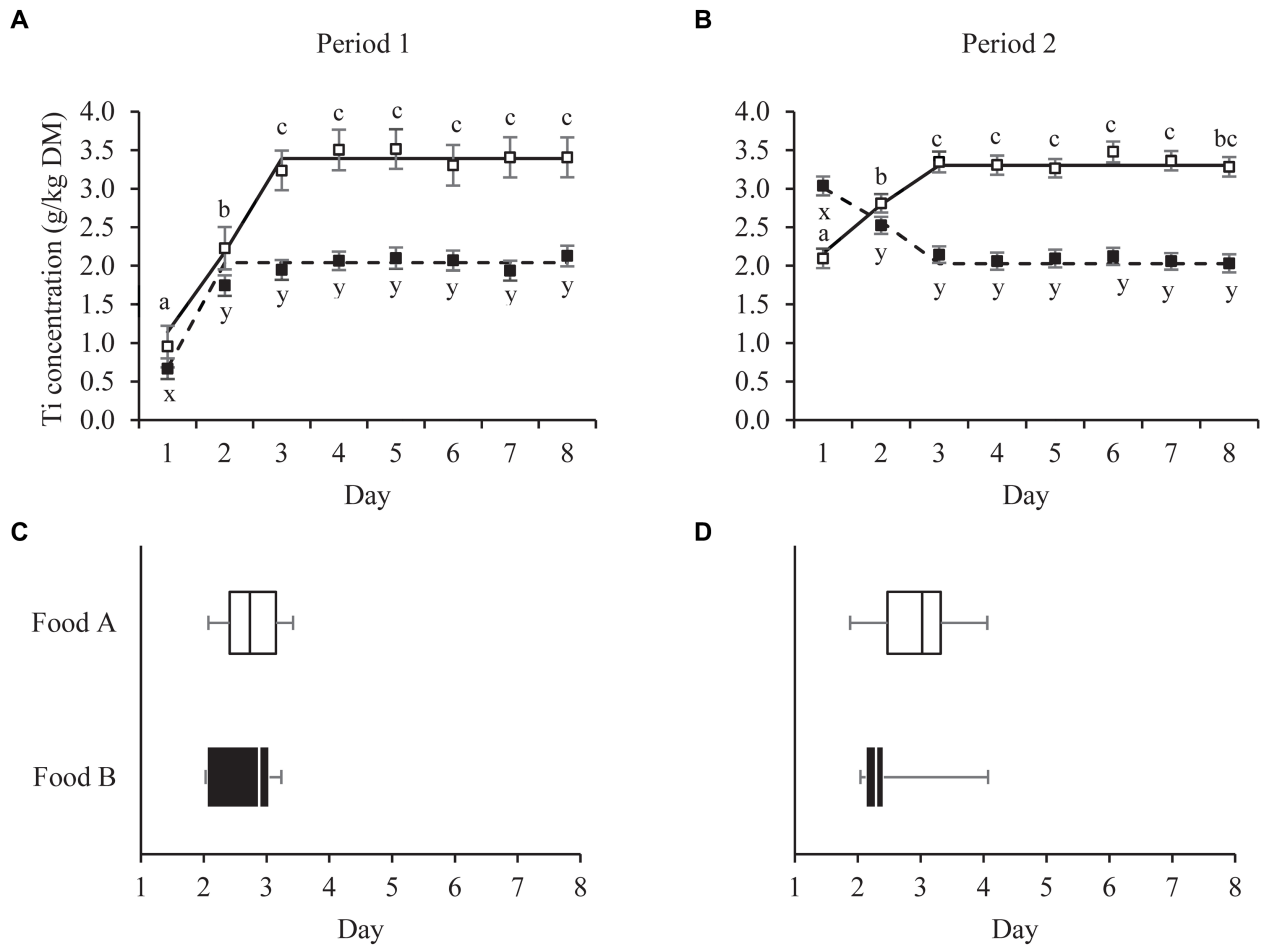
Mean daily fecal titanium (Ti) concentrations (panel **A,B**) of pet cats fed a relatively high (Food **A**; □) and low (Food B; ■) digestible food over two consecutive 8-day periods (P1, P2). Broken-line analysis showed fecal Ti concentrations to plateau at day (mean ± SE) 3.17 ± 0.24 (□, P1, *n* = 12), 2.81 ± 0.39 (□, P2, *n* = 14), 2.28 ± 0.17 (■, P1, *n* = 14) and 3.14 ± 0.26 (■, P1, *n* = 12). Means within panel (**A,B**) and food (□, ■) with different superscripts (a,b,c or x,y,z) differ (*p* < 0.05). Error bars are standard errors of the mean. Variation in breakpoint values for individual cats are shown in panel **C** and **D** (□, P1, *n* = 10; □, P2, *n* = 14; ■, P1, *n* = 12; ■, P2, *n* = 9). Box plots represent the lower quartile, median and upper quartile, the whiskers extend to the minimal and maximal values.

Ti concentrations in the feces stabilized around the third day after first food consumption. The breakpoints in the fecal Ti concentration of all cats fed Food A were at day 3.17 (*n* = 12) for period 1 and 2.81 (*n* = 14) for period 2 (Figure 1). For all cats fed Food B, the breakpoints were estimated at day 2.28 (*n* = 14) for period 1 and 3.14 (*n* = 12) for period 2. The breakpoints for the individual cats ranged between 1.81–4.05 (Food A, period 1, *n* = 10), 2.07–3.42 (Food A, period 2, *n* = 14), 2.04–4.07 (Food B, period 1, *n* = 12) and 2.03–3.23 (Food B, period 2, *n* = 9) (Figure 1). The findings were supported by repeated measures analyzes of variance on the same data sets, which showed that fecal Ti concentrations in cats fed Food A (in both feeding periods) increased from day 1 to 2 (*p* < 0.001) and from day 2 to 3 (*p* < 0.05), with no differences from day 3 onwards (Figure 1). Fecal Ti concentrations in cats fed Food B differed from day 1 to 2 in both feeding period 1 (*p* < 0.001) and 2 (*p* < 0.05). For cats fed Food B there were no significant differences already from day 2 onwards, during both feeding periods, but in period 2 fecal Ti concentrations on day 2 tended to differ from those on days 3 (*p =* 0.077), 4 (*p =* 0.052), 7 (*p =* 0.065) and 8 (*p =* 0.056).

### 3.4. Variation in fecal apparent digestibility values across days

**Figure 2:**
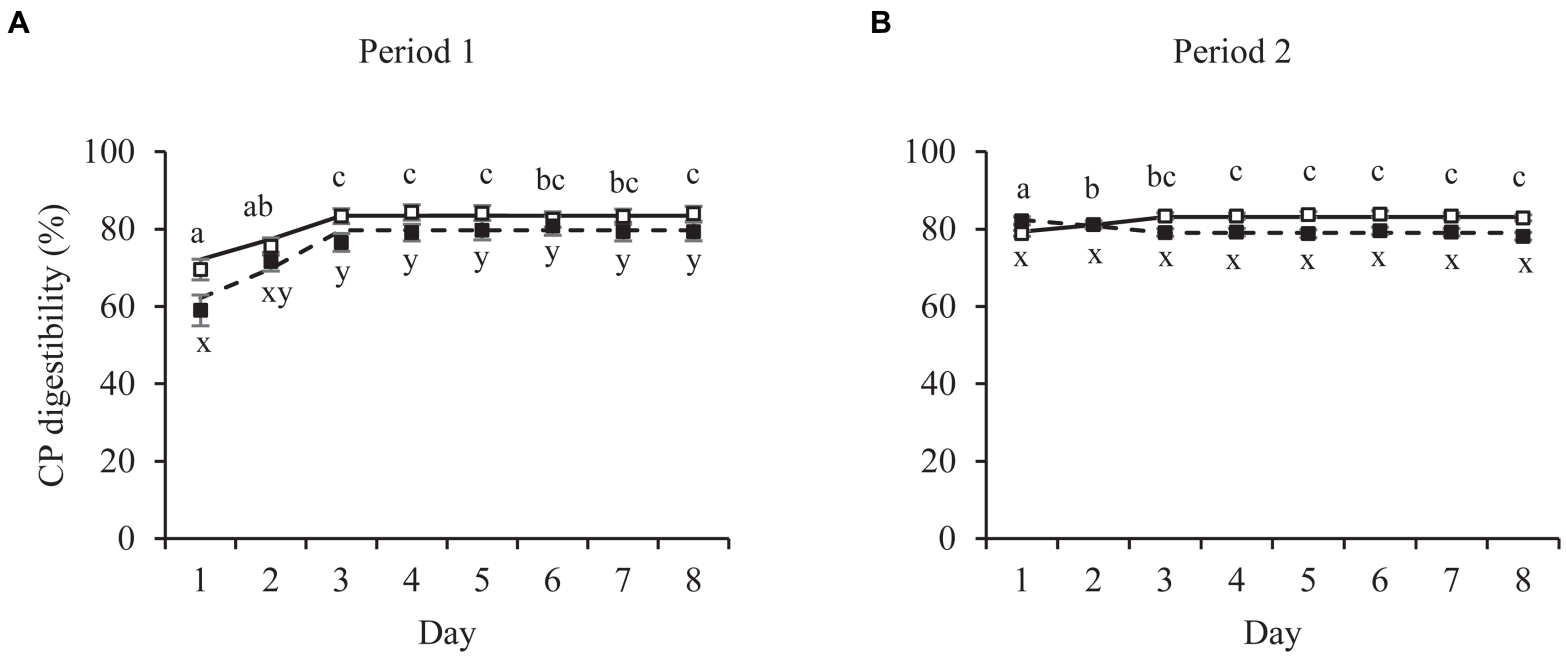
Mean daily crude protein (CP) fecal apparent digestibility values of pet cats fed a relatively high (□) and low (■) digestible food over two consecutive 8-day periods (P1, P2). Broken-line analysis showed CP digestibility values to plateau at day (mean ± SE) 3.15 ± 0.36 (□, P1, *n* = 12), 3.21 ± 0.58 (□, P2, n = 14), 3.30 ± 0.37 (■, P1, *n* = 14) and 3.20 ± 0.69 (■, P2, *n* = 12). Means within panel **(A,B)** and food (□, ■) with different superscripts (a,b,c or x,y) differ (*p* < 0.05). Error bars are standard errors of the mean.

Similar patterns over time were present for apparent fecal digestibility values (Figure 2) to those of fecal Ti concentrations. The broken-line analyzes on the daily averages per food and period resulted in breakpoints ranging from 3.15 ± 0.36 for CP (Food A, period 1) to 3.63 ± 0.87 for Cfat (Food A, period 1; Table 2), except for Cfat digestibility values of cats fed Food B (breakpoint of 4.58 ± 0.92). The outcomes of the repeated ANOVA of the same datasets were in line with those of the linear broken-line regressions. Digestibility values for cats fed Food A in period 1 were constant from day 3 onwards for CP (Figure 2), DM and GE (Supplementary Figure S2), with no day effects for Cfat (Supplementary Figure S2). During period 2, digestibility values of cats fed Food A were constant from day 2 onwards for Cfat and from day 3 onwards for DM, CP and GE. Digestibility values for cats fed Food B were constant from day 2 onwards for period 1. In period 2, this was from day 1 onwards for CP, from day 2 for GE and from day 3 for DM and Cfat.

**Table 2:** Pet cats (*n* = 26) received 2 test foods for 2 subsequent 8-day periods. Digestibility values were calculated from daily fecal samples and analyzed for time effects by means of linear broken-line regressions. Presented are the estimated breakpoints in days ± standard error of fecal titanium (Ti) concentration and daily digestibility values of dry matter, crude protein, crude fat, and gross energy, separately for the first or second trial (period) and high digestible food (A) or low digestible food (B).

	Breakpoint	
	Food A	Food B
*Period 1*	*n* = 12	*n* = 14
Titanium	3.17 ± 0.24	2.28 ± 0.17
Dry matter	3.21 ± 0.32	3.35 ± 0.41
Crude protein	3.15 ± 0.36	3.30 ± 0.37
CfatCrude fat	3.63 ± 0.87	1.86 ± 0.16
Gross energy	3.17 ± 0.32	3.37 ± 0.43
*Period 2*	*n* = 14	*n* = 12
Titanium	2.81 ± 0.39	3.14 ± 0.26
Dry matter	2.53 ± 0.18	3.26 ± 0.33
Crude protein	3.21 ± 0.58	3.20 ± 0.69
Crude fat	3.09 ± 0.44	4.58 ± 0.92
Gross energy	2.67 ± 0.21	3.24 ± 0.34

### 3.5. Number of fecal collection days and sample size

**Figure 3:**
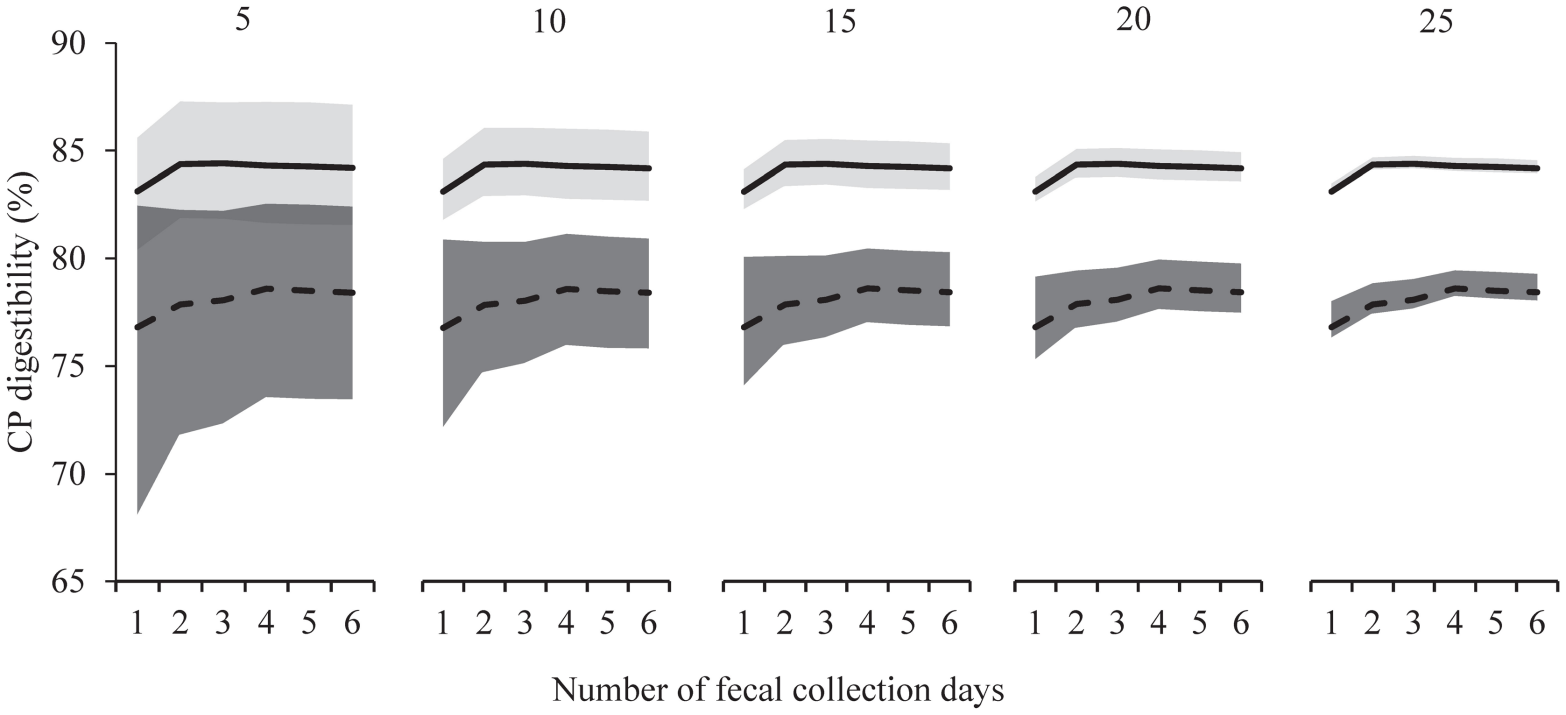
Bootstrapped estimates and confidence intervals of crude protein (CP) fecal apparent digestibility for the relatively high digestible food (Food A, upper solid line, light grey area) and relatively low digestible food (Food B, lower dashed line, dark grey area) with increasing number of fecal collection days (1 to 6, x-axis). Day one represents the first stable fecal collection day (i.e., trial day 3) with 3 to 6 days representing mean values from consecutive collection days (trial days 3 to 8, *n* = 26 cats). Bootstrap sampling included 10,000 replicates and were ran for different sample sizes (5, 10, 25).

The use of multiple subsequent days, as opposed to fecal samples of day 3 only, did not decrease variation in the digestibility estimates. Bootstrap analyzes compared data from only day 3 to those for multiple days created by pooling (i.e., days 3–4 up to days 3–8), and the inclusion of multiple days did not reduce confidence interval width (Figure 3; Supplementary Figure S3). In the scenarios of increasing the number of cats, bootstrap analyzes showed decreasing variation and reductions in confidence interval width, both for Foods A and B (Figure 3; Supplementary Figure S3).

### 3.6. Experimental factors and cat characteristics

Digestibility values for all nutrients were higher in Food A compared to Food B (all nutrients *p* < 0.001, but Cfat *p* < 0.05), and unaffected by the test period and the cat characteristics sex, neuter status, and body weight (*p* ≥ 0.05; Table 3). Significant interaction effects in Cfat digestibility showed that male cats had lower digestibility values compared to female cats, only when fed Food B (2-way interaction sex × food *p* = 0.003). Male cats showed a relatively steep increase in Cfat digestibility with increasing weight (sex × body weight *p* = 0.013) and a steeper decrease with increasing age (sex × age *p* = 0.002). Increases in Cfat digestibility with increasing body weight were most pronounced when Food B was fed (food × body weight *p* = 0.022). GE digestibility values in males decreased more strongly with age than those in females (sex × age *p* = 0.019). Main effects part of significant interactions are not addressed here as interactions provide the more accurate effects.

**Table 3:** Apparent fecal digestibility values (%) were determined in 26 pet cats that received 2 different foods (high digestible Food A and low digestible Food B) during 2 subsequent 8 day periods. The digestibility values for days 3–8, i.e., after values stabilized, were analyzed with repeated measures ANOVA for effects of test period, test food, and the cats’ sex, neuter status, body weight and age. The latter two appeared to be non-significant and are not reported below. Presented are the least square means ± standard error of dry matter, crude protein, crude fat, and gross energy.

Factor	Dry matter	Crude protein	Crude fat	Gross energy
Period				
1 (*n* = 26)	n.i.	n.i.	n.i.	n.i.
2 (*n* = 26)				
Food				
A (*n* = 26)	83.3 ± 0.4^a^	83.0 ± 0.5^a^	91.5 ± 0.6^a^	85.5 ± 0.4^a^
B (*n* = 26)	74.6 ± 0.4^b^	78.4 ± 0.5^b^	87.8 ± 0.6^b^	77.6 ± 0.4^b^
Sex				
Female (*n* = 16)	n.i.	81.4 ± 0.5	92.1 ± 0.6	82.8 ± 0.5
Male (*n* = 10)		80.0 ± 0.6	87.3 ± 0.8	80.3 ± 0.6
Neuter status				
Intact (*n* = 3)	n.i.	n.i.	n.i.	n.i.
Neutered (*n* = 23)				
Food*Sex				
*A -Female*	n.i.	n.i.	92.4 ± 0.8^a^	n.i.
*A -Male*			90.6 ± 1.0^a^	
*B -Female*			91.7 ± 0.8^a^	
*B -Male*			83.9 ± 1.1^b^	

n.i., Not included in the analysis of variance after model selection using stepwise regression. ^a,b^Means differ significantly (within independent variable and nutrient) when they do not share any superscript letter (*p* < 0.05).

## 4. Discussion

In-home food digestibility trials with privately-owned cats have the potential to produce highly representative data for the target population, but currently there are no validated protocols. The present study is unique for determining cat food digestibility in-home and we analyzed variation in digestibility values to establish the minimal number of days for adaptation and fecal collection required for reliable apparent fecal digestibility estimates of dietary nutrients and energy. Also, we analyzed the impact of the study cat population, in terms of sample size and cat characteristics, on digestibility estimates in order to determine the minimal number of animals required for an *a priori* set acceptable margin of error.

### 4.1. Adaptation period

Food digestibility measurements with an indigestible marker require a sufficiently long adaptation period to ensure a constant rate of marker excretion in the feces, as well as to allow the adaptation of food-specific digestive processes (incl. gut motility, enzyme secretions, absorption, microbial fermentation, etc.).

### 4.2. Constant fecal marker excretion

This study shows that fecal samples from day 3 onwards can be used for the determination of apparent fecal digestibility values. Linear broken-line regressions revealed a plateau in the cats’ fecal Ti concentrations from on average days 3.17 (period 1) and 2.82 (period 2) onwards for the more digestible food (Food A) and from respective days 2.28 and 3.14 onwards for the less digestible food (Food B). Repeated measures analyzes of variance confirmed that Ti concentration in fecal samples from cats fed the more digestible food (Food A) collected on day 3 (i.e., the interval of 48–72 h) did not differ (*p* ≥ 0.10) from those in samples collected the following days, in both feeding periods. For cats fed the less digestible food this was true already (*p* ≥ 0.05) on day 2 (i.e., the interval of 24–48 h).

Owners in the current study collected feces from the cats’ litterbox on a daily basis. Although the time of feces collection was recorded, the precise defecation time was unknown. Feces collected during day 1 were produced between 0–24 h after the first morning meal, and therefore labelled as the average time point of 12 h post first food consumption. Subsequent days were labelled similarly. As such, day 2.28 represented 42.7 h (2.28 × 24 h – 12 h), whereas day 3.17 represented 64.1 h (3.17 × 24 h – 12 h) post first food consumption. Therefore, findings indicate that fecal samples from day 3 onwards can be used for the determination of apparent fecal digestibility values, which is 1 day later than was found in our similar in-home digestibility study with 53 privately-owned dogs (8).

In the in-home study with dogs, 1 day was sufficient to reach stable fecal marker (Ti) concentrations for both a relatively high and a low digestible dog food (8). In cats, peak marker (chromium oxide) concentrations in feces have been reported at (mean ± std) 26.5 ± 5.8 h post meal consumption in 3 year-old cats (*n* = 6) and 35.7 ± 14.1 h in 11 year-old cats (*n* = 6), as determined in dedicated feline research facilities (15). The latter study did not demonstrate significant differences between age groups, but the total transit times were highly variable across individuals, especially in the older cats. In the present study, breakpoints estimated for individual cats ranged between day 1.81 to 4.07, across foods and feeding periods, indicating the necessity to account for individual variation in transit time as a determinant of the time to reach stable fecal marker concentrations.

Gastrointestinal transit time can be influenced by several factors including dietary fiber content and source, the amount of food in the gastrointestinal tract, and the gastric emptying rate. A diet high in fiber generally decreases transit time and cats receiving a diet including 10% cellulose had a gastrointestinal transit time of 15.2 h compared to 20.6 h for cats receiving a diet without cellulose (38). Similarly, cats receiving a diet with 16% fiber-rich beet pulp had a transit time of 15.2 h compared to 21.5 h for cats who did not receive beet pulp (39). In the present study, foods differed slightly in the time at which the fecal marker excretions were constant (*p* < 0.05), with 2 days for the more digestible food with 103 g/kg total dietary fiber and 1 day for the less digestible food with 191 g/kg total dietary fiber.

In addition to fiber content and source, the amount of food present in the gastrointestinal tract influences the gastrointestinal transit time. A large meal size reduces gastrointestinal transit by reducing gastric emptying (16). The gastrointestinal transit times of cats (*n* = 6) prior and post feeding, determined by a capsule, were reported to be 28.9 h (range 18.4–90.9 h) and 46.6 h (range 15.0–109.4 h), respectively (14). The present study included cats with different consumption patterns, with 10 cats being normally fed meals and 17 *ad libitum*, but nevertheless shows 1–2 days to be sufficient to reach a stable fecal marker excretion, depending somewhat on the test food. Considering the importance of stable fecal marker excretions for accurate digestibility estimation, it is relevant to further study gastrointestinal transit times in cats for more extreme food contrasts such as wet versus dry food, food formulations, compositions, and feeding regimes.

### 4.3. Digestive adaptation

This study shows that the cats’ digestive system appeared to adapt rapidly to novel foods and an adaptation period of 5 days as indicated by AAFCO (40) and FEDIAF (41) can be shortened to 2 days to yield stable digestibility values. An adaptation period in food digestibility studies should ensure that a cat’s digestive system can adapt to the test food to achieve a steady state. Digestive adaptation, such as adjustments in digestive enzyme activity and gut microbiota composition, occur already within a few hours after changes in the amount of dietary protein, carbohydrate or lipid, in humans and several animal species (9, 11, 42). Digestive adaptation of the cats in the present study apparently required a maximum of 2 days as all nutrient apparent digestibility values were constant from a maximum of day 3 onwards. For the less digestible Food B, this time included 2 days for all nutrients in period 1 and 1 day for CP apparent digestibility values in period 2. The differences in time to reach stable digestibility values (Figure 2; Table 2; Supplementary Figure S2) between CP, DM, Cfat and GE indicate different adaptation processes/mechanisms. Compared to dogs (8), cats need 1 day longer to adapt, at least regarding Ti, CP, DM, Cfat and GE, which might be attributed to a less regular defecation pattern compared to dogs. The difference in ME requirements between dogs and cats (41), translates in higher amounts of food consumed by dogs compared to cats if foods with comparable ME contents are fed. A 4 to 7-y.o. dog with moderate activity consumes about 110 kcal ME/kg BW^0.75^ whereas an adult cat (4 kg) consumed between 57 (neutered/indoor) to 88 (active) kcal ME/kg BW^0.75^ (41). In case dogs and cats have largely similar digestive efficiencies (43–45), dogs would then also have a higher mass of undigested matter that is excreted via the feces than cats. In the present study, cats defecated on average 1.2 times/d whereas this was 2.3 times/d for dogs (*n* = 53) fed dry foods in our previous study (8). The average fresh fecal weights also differed between dogs (4.1 g/kg BW^0.75^) and cats (5.9 g/kg BW^0.75^). Cats have profound lower feeding levels, defecated almost half as frequent as dogs and with larger fecal volumes per kg metabolic body weight, which underlies differences in adaptation periods as well as opportunities for fecal collection for in-home digestibility measurements.

### 4.4. Fecal collection period

Current guidelines recommend 5 fecal collection days for cat food digestibility studies with the marker method (41), although the scientific support for this period is unclear. In-home food digestibility trials are expected to have a relatively larger measurement variation, warranting more sample collection days, given the less controlled conditions and sources of variation such as consumption of other matter (e.g. treats, food) or contamination of feces (e.g. cat litter). Nevertheless, digestibility values in the present in-home study were stable from days 3 onwards, and 1 day of fecal collection seemed to suffice for a precise dietary digestibility determination of CP, DM, Cfat and GE. The pooling of fecal samples, which we modelled by combining results from 2 up to 6 fecal collection days, did not significantly decrease confidence interval width (Figure 3; Supplementary Figure S3). Additional fecal collection days do not increase precision substantially in food digestibility trials that make use of a marker, similar to what was found in the in-home study with dogs (8). Practicing multiple fecal collection days, however, does have the advantage of being able to deal with infrequent defecations or small fecal volumes.

### 4.5. Study population

The appropriate number of cats for in-home digestibility studies relates strongly to variation in digestibility values and accepted margin of error of the digestibility assessment. The margin of error is described as half of the 95% confidence interval around the mean and was, on average 2.4% for both DM and CP, and 2.1% for GE, in apparent fecal digestibility trials with dry foods conducted following the standard AAFCO quantitative collection protocol at cat research facilities in the US (*n* = 6 cats per food, 129 foods from Hall et al. (46) (Hall personal communication)). Similar values of 2.9% for DM (range 0.8–4.7%), 2.5% for CP (1.0–4.0%) and 2.4% for GE (0.8–3.9%) were found at a research facility in Brazil (12 studies, 6 cats/food; Carciofi personal communication). The ranges in the latter studies indicate that the margin of error currently accepted for digestibility testing varies per test but also per nutrient of interest. The required number of cats for in-home digestibility testing can be based on these averages from Hall et al. (46) and maximal margin of errors from Carciofi. For the more digestible food this would result in a required sample size of <5 for DM, <5 to 6 for CP and < 5 for GE. For the less digestible food this would be <5 to 6 cats for DM, 8 to 12 for CP and < 5 to 7 for GE (Supplementary Figure S4). These two test foods were formulated to differ in composition and digestibility, but did not cover the full range as exists in commercial cat foods. For example, the crude protein content of test foods A and B were, respectively, 277 and 287 g/kg DM and apparent fecal crude protein digestibility values were 83 and 78%, whereas in complete dry cat foods the protein content can range from 234 to 489 g/kg DM (47) and the apparent fecal crude protein digestibility from 78 to 94% (20, 48–51). Future in-home digestibility trials with cat foods that range in composition and digestibility can further specify the required sample sizes.

Required sample sizes for in-home food digestibility trials depend on the test food used and the variation in digestibility values that originates from test subjects and study conditions (e.g., owner compliance and fecal sample contamination). The possible influence of cat characteristics on food digestibility values showed here as males having lower Cfat digestibility values when fed Food B, a steeper increase in Cfat digestibility values with increasing weight, and a steeper decrease in Cfat and GE digestibility values with increasing age, all compared to females (significant 2-way interactions with sex). In addition, the increase in Cfat digestibility values with increasing weight was seen across sexes, but only when fed Food B. The sex of a cat is a known influence on the digestion of food for example through effects on lipid metabolism. In a comparison between neutered lean and obese males (*n* = 10) and females (*n* = 10), the male cats were more sensitive to insulin for fatty acid uptake, especially when they were obese (52). Age too is well-known for influencing digestibility values in cats and aging decreases digestibility values for dry matter, protein, fat and energy (18, 20, 23, 53), potentially due to a reduced secretion and activity of digestive enzymes and a reduced capacity for the production, transport, and secretion of bile acids (54). Thus, a multitude of cat-related factors, separately or through interaction, potentially affect food digestibility measurements, causing variation and larger sample sizes required for heterogenous study populations. The advantage a heterogenous group of pet cats with varying characteristics is the good representativeness of study outcomes for a heterogenous target pet population.

### 4.6. Application for future in-home testing

In-home digestibility testing of cat food has the potential to produce data that are more representative for the pet cat population these foods are intended for than those currently derived from dedicated cat research facilities. Information on apparent nutrient digestibility is collected from cats with different characteristics (e.g. sex, neuter status, age, breed, food history) and living conditions (e.g. housing, eating pattern, activity), and additional information on perceived food quality characteristics can be obtained from the owner. For the realization of the in-home test potential, owner compliance is of crucial important. Non-compliance, like through the (accidental) provision of additional food or treats or fecal sample contamination (e.g. with cat litter), increases variation in digestibility values and can lead to misinterpretations. Owner compliance is influenced by the duration and complexity of the requested tasks (55) and designing studies of minimal length is of high relevance. Compliance may be further facilitated by short training programs to familiarize cat owners with the tasks and instruct them on study aspects such as fecal scoring (56). Future studies may assess the precise impact of non-compliance on digestibility values.

Indigestible markers are practical for pet food digestibility measurements in a home environment and TiO_2_ has been validated as a digestibility marker for multiple animal species (3, 57–63). It has been used extensively in the past, but recently it was considered to be unsafe as a feed additive (64, 65), due to the potential of nanoparticles to be absorbed in the body. The TiO_2_ used in the present study did not contain nanoparticles defined by a particle size <100 nm^29^ and ingested concentrations (0.02 g/kg BW) were below the no-adverse effect level of 1 g/kg body weight. However, to address future concerns of owners regarding the safety of test foods, alternative markers should be investigated and made available for in-home digestibility studies.

The present digestibility values were not compared to those in cats at dedicated research facilities. We do not consider the latter as a benchmark for privately owned cats and presume that study outcomes are partly specific for the test conditions (research facilities vs. in-home). Further studies could focus on identifying and controlling sources of variation for in-home digestibility testing which will lead to improved repeatability, accuracy, and precision thereby making in-home testing more attractive for routine use in pet food testing.

## 5. Conclusion

In-home cat food digestibility trials require validated test protocols and this study sets key trial variables and also sheds new light on the digestibility test protocols currently used in cat research facilities. The findings support that 2 adaptation days and 1 fecal collection day suffice for accurate digestibility estimates. Three fecal collection days are recommended though, as to account for the irregular defecation pattern of the cat. Assuming a margin of error currently accepted for digestibility testing, the required sample size for an in-home food test of digestibility would range from 5 to 12 cats depending on properties of food and nutrient of interest. Sources of variation in digestibility values are cat characteristics like age and sex, and owner compliance, which both warrant further investigation.

## Data availability statement

The raw data supporting the conclusions of this article will be made available by the authors, without undue reservation.

## Ethics statement

Ethical review and approval was not required for the study on human participants in accordance with the local legislation and institutional requirements. The patients/participants provided their written informed consent to participate in this study.

The animal study was reviewed and approved by Animal Welfare Body of Wageningen University (Wageningen, Netherlands). Written informed consent was obtained from the owners for the participation of their animals in this study.

## Author contributions

EB, WH, BB, and GB contributed to the design of the study and interpretation of the findings. Execution of the study and chemical analyzes were done by EB. Data processing and analyzing were done by EB, GB, and BB. The manuscript has been written by EB and revised by GB, BB, and WH. All authors contributed to the article and approved the submitted version.

## Funding

This research was supported by University Fund Wageningen (project 20190501; In-Home testing of Petfood).

## Conflict of interest

The authors declare that the research was conducted in the absence of any commercial or financial relationships that could be construed as a potential conflict of interest.

## Publisher’s note

All claims expressed in this article are solely those of the authors and do not necessarily represent those of their affiliated organizations, or those of the publisher, the editors and the reviewers. Any product that may be evaluated in this article, or claim that may be made by its manufacturer, is not guaranteed or endorsed by the publisher.
