# Supplementary material for: Determining the protocol requirements of in-home cat food digestibility testing
Source: Front Vet Sci. 2023 May 11;10:1129775. doi: 10.3389/fvets.2023.1129775 (PMC10213761; doi:10.3389/fvets.2023.1129775)
Supplement: Supplementary file 1 [file Data_Sheet_1.docx]

***Supplementary Material***

**Determining the protocol requirements of in-home cat food digestibility testing**

**E. Bos*, W.H. Hendriks, B. Beerda, G. Bosch**

*** Correspondence:** E.Bos: [evelien.bos@wur.nl](mailto:evelien.bos@wur.nl)

Cat is not eligible

Cat is not eligible

Owner withdraws

Cat does not eat both foods

Owner shows severe non-compliance

Applied for participation

Selected for participation

Contact established

Started the trial

Finished the trial

n = 107

Owner is not eligible

n = 55

No response

n = 30

n = 27

Cat tastes food samples

n = 9

Cat does not eat both test foods

**Figure 1.** Flowchart of participation of cats (n) from owners applying to participate, to the number of cats finishing the trial and reasons for exclusion in the various stages of the recruitment/trial.

**Table 1.** Characteristics of participants that were included in the data analyses.

| **Category** | **Level/range** | **n** |
| --- | --- | --- |
| *Cats* |  | *26* |
| Sex | Males  Females | 10  16 |
| Neuter status | Intact  Neutered | 3  23 |
| Age (years) | 1-16 | - |
| Weight (kg) | 2.9-7.5 | - |
| Body condition score^1^ | 1  2  3  4  5 | 0  2  18  6  0 |
| Fur length | Short-haired  Long-haired | 22  4 |
| Breed | European shorthair  Other breed  Mixed breed  Unknown | 12  4  4  6 |
| Prior food type | Kibbles  Canned  Kibbles + canned | 20  1  5 |
| Food history (number of prior foods) | 1-2  3-5  >5 | 7  5  14 |
| Usual food pattern | *Ad libitum*  Meals  *Ad libitum* + meals | 15  9  2 |
| *Cat owners* |  | *25* |
| Age (years) | <20  20-29  30-39  40-49  50-59  60-69  ≥70 | 0  10  6  3  4  2  0 |
| Gender | Males  Females | 2  23 |
| Education | Secondary education  Primary vocational education  Secondary vocational education  Higher vocational education  Scientific education | 4  1  5  7  8 |
| Presence of other cats | Yes  No | 3  22 |
| Presence of other pets | Yes  No | 10  15 |
| Presence of children | Yes  No | 5  20 |
| Number of adults | 1  2  3  4  >4 | 9  11  2  1  2 |

^1^Derived from FEDIAF (2020).

**(B)**

**(A)**

Period 2

Period 1

x

c

c

c

c

c

c

b

c

c

c

c

c

c

b

a

yz

yz

yz

yz

yz

y

z

a

y

y

y

y

y

y

xy

x

**(D)**

**(C)**

xy

x

b

b

b

b

b

b

yz

yz

yz

z

yz

yz

b

a

y

y

y

y

y

y

y

x

**(F)**

**(E)**

x

c

c

c

c

c

c

b

b

a

b

b

b

b

b

y

y

y

y

y

y

y

a

y

y

y

y

y

y

xy

a

x

**Figure 2.** Mean daily fecal apparent digestibility of dry matter (DM; panel **A**, **B**), crude fat (Cfat; panel **C**, **D**) and gross energy (GE; panel **E**, **F**) of pet cats fed a relatively high (□) and low (■) digestible food during two consecutive periods (P1, panel **A**, **C**, **E**; P2, panel **B**, **D**, **F**) of eight days. Cats were fed the foods in a cross-over study (n=12 and 14 during P1 for food □ and ■, respectively). Means within panel and food (□, ■) with different superscripts (a,b,c or x,y,z) differ (*P*<0.05). Error bars are standard errors of the mean.

**(A)**

5

10

15

20

25

Number of fecal collection days

5

10

15

20

25

**(B)**

**(C)**

Number of fecal collection days

5

10

15

20

25

Number of fecal collection days

**Figure 3.** Bootstrapped estimates and confidence intervals of dry matter (**A**), crude fat (**B**) and gross energy (**C**) fecal apparent digestibility of two dry foods (A, □; B, ■) with increasing number of fecal collection days (1 to 6) and cats (5 to 25). Bootstrap sampling included 10,000 replicates. One day represents the first accurate fecal collection day (day 3 after feeding food A or B) with 3 to 6 days representing calculated values from the addition of subsequent collection days (days 3 to 8).

Gross energy

Crude protein

Dry matter

Number of cats

**Figure 4.** The margin of error of the bootstrapped estimates of dry matter, crude protein and gross energy for a relatively high (□) and low (■) digestible food with an increasing number of cats. The margin of error equals half of the 95% confidence interval. The average and maximal margin of error are as found in kennel tests, adapted from Hall et al. (2013) and data from Dr. Carciofi (personal communication).
